# Individual differences in cognitive performance and brain structure in typically developing children

**DOI:** 10.1016/j.dcn.2015.05.003

**Published:** 2015-05-21

**Authors:** Susumu Yokota, Hikaru Takeuchi, Teruo Hashimoto, Hiroshi Hashizume, Kohei Asano, Michiko Asano, Yuko Sassa, Yasuyuki Taki, Ryuta Kawashima

**Affiliations:** a Division of Developmental Cognitive Neuroscience, Institute of Development, Aging and Cancer, Tohoku University, 4-1 Seiryo-cho, Aoba-ku, Sendai 980-8575, Japan; b Division of Medical Neuroimaging Analysis, Department of Community Medical Supports, Tohoku Medical Megabank Organization, Tohoku University, 4-1 Seiryo-cho, Aoba-ku, Sendai 980-8575, Japan; c Department of Nuclear Medicine & Radiology, Institute of Development, Aging and Cancer, Tohoku University, 4-1 Seiryo-cho, Aoba-ku, Sendai 980-8575, Japan; d Smart Ageing International Research Centre, Institute of Development, Aging and Cancer, Tohoku University, 4-1 Seiryo-cho, Aoba-ku, Sendai 980-8575, Japan; e Department of Functional Brain Imaging, Institute of Development, Aging and Cancer, Tohoku University, 4-1 Seiryo-cho, Aoba-ku, Sendai 980-8575, Japan

**Keywords:** MRI, Wechsler Intelligence Scale for Children, Factor index scores, Cognitive patterns, Voxel-based morphometry, Gray matter volume

## Abstract

Individual differences in cognitive patterning is informative in understanding one's cognitive strengths and weaknesses. However, little is known about the difference in brain structures relating to individual differences in cognitive patterning. In this study, we classified typically developing children (*n* = 277; age range, 5–16 years) into subtypes with k-means cluster analysis along with factor index scores using the Wechsler Intelligence Scale for Children (Third Edition). We then applied voxel-based morphometry to investigate whether significant gray-matter-volume differences existed among subtypes of cognitive patterns. Depending on the level of performance and cognitive patterning, we obtained six subtypes. One subtype that generally scored below average showed larger volume in the right middle temporal gyrus than the other five. On the other hand, two subtypes that achieved average levels of performance showed reverse-patterned factor index scores (one scored higher in Verbal Comprehension and Freedom from Distractibility, and the other scored lower in these two factor index scores) and had smaller volume in the right middle temporal gyrus than the other subtypes. From these results, we concluded that cognitive discrepancy was also obvious in typically developing children and that differences in cognitive patterning are represented in brain structure.

## Highlights

• We investigated the relationship between cognitive pattern and gray matter volume.
• Cognitive patterns were classified into 6 subtypes.
• Volume of right middle temporal gyrus was differentiated by cognitive patters.

## 1 Introduction

The intelligence quotient (IQ) has played a significant role in clinical psychology. The Wechsler Intelligence Scale for Children (WISC) is one of the most widely used measures of psychometric intelligence in children. It is used in various contexts such as clinical, academic, and school settings. This instrument has excellent psychometric properties, especially in terms of its standardization and the reliability of the full-scale IQ (FSIQ), two composed scores that are verbal IQ (VIQ), performance IQ (PIQ), and four factor index scores (VC: verbal comprehension, PO: perceptual organization, FD: freedom from distractibility, and PS: processing speed) (Kaufman, 1993). Some practitioners have reported the importance of the profile analysis of factor index scores or sub-test scores with respect to patterning (Donders, 1996). The discrepancy pattern of cognitive functions measured by WISC is useful for better understanding the cognitive strengths and weaknesses of children.

In psychology, previous studies have categorized individual cognitive patterns using factor index scores in typically developing children (Donders, 1996, Glutting et al., 1994), as well as in clinically referred children or children with neurodevelopmental disorders, such as autism, traumatic brain injury, or attention deficit and hyperactivity disorder (ADHD) (Allen et al., 1991, Donders and Warschausky, 1997, Hale et al., 2013, Mayes and Calhoun, 2004, Mayes and Calhoun, 2006, Siegel et al., 1996, Waxman and Casey, 2006). The number of subtypes has been varied from 3 to 8 according to the characteristics of participants or versions of the WISC. Glutting et al. (1994), who investigated the pattern of factor index scores combined with the Wechsler Individual Achievement Test of composite scores, revealed six subtypes within 824 typically developing children and found that the six subtypes were characterized by general performance level (above-average, average, and below-average) and the discrepancy of sub-scores. Similarly, Donders (1996) clarified five subtypes within 2200 children from WISC-III standardization samples. Three out of the five subtypes were featured by the performance levels (above-average, average, and below-average overall performance levels), and the remaining two were featured by performance patterns. The results of Glutting et al. (1994) and Donders (1996) suggest that cognitive patterns can be classified into subtypes on the basis of general performance level and the discrepancy of factor index scores, even in typically developing children.

The relationship between brain structure and IQ has been reported by several previous studies. Regarding the neural basis of cognitive functions, several studies have found the correlation between composite scores of the WISCs or Wechsler Adult Intelligence Scales (WAISs) and brain size and between volume or thickness of cortical areas and white matter integrity in normal populations (Burgaleta et al., 2014, Haier et al., 2004, Jung and Haier, 2007, Lee et al., 2006, Sowell et al., 2001, Sowell et al., 2004). Willerman et al. (1991) examined the relationship between brain size and intelligence, demonstrating positive correlation between IQ scores and brain size. Wilke et al. (2003) investigated 146 children (age range: 5–18 years), revealing a tendency for the correlation between FSIQ and global gray matter volume and regional gray matter volume (rGMV) in the anterior cingulate cortex. Frangou et al. (2004) examined 40 young participants (mean age of 14.26 years), revealing a significant positive correlation between FSIQ and rGMV in the cerebellum, orbitofrontal cortex, thalamus, cingulate, and precuneus and a significant negative correlation in the caudate nucleus. Haier et al. (2004) found correlations among rGMV within frontal (Brodmann areas (BA) 10, 46, 9), temporal (BA 21, 37, 42), parietal (BA 43, 3), and occipital (BA 19) lobes in adolescents and adults. From the results of previous findings, rGMV and the frontal, temporal, and parietal regions are positively correlated with FSIQ. Furthermore, the previous studies on cortical thickness revealed that brain regions that positively correlated with FSIQ include areas in the frontal (inferior, anterior, or superior), temporal (anterior, superior), and parietal cortices (Burgaleta et al., 2014, Choi et al., 2008, Karama et al., 2013, Margolis et al., 2013, Narr et al., 2007).

Previous brain structure and function studies mainly focused on FSIQ as cognitive function. However, little is known about the neural basis of the discrepancy of cognitive functions among individuals. Considering the taxonomy of cognitive patterning in the normal population, brain structure was considered to differ among subtypes. Moreover, the number of participants in previous imaging studies was relatively small. Therefore, in this study, we examined the relationship between brain structure and cognitive subtypes of typically developing children obtained from a large sample. Identifying the relationship between brain structure and cognitive pattern would help to explore the biological underpinnings of intelligence and the specific cognitive patterns associated with neurodevelopmental disorders.

We hypothesized that the subtypes of cognitive pattern emerged depending on the performance levels (above-average, average, and below-average) and performance patterns. Brain structures previously associated with IQ, including the prefrontal, temporal, and parietal cortex, were significantly related to certain cognitive profile subtypes.

## 2 Methods

### 2.1 Participants

We collected brain magnetic resonance (MR) images from 298 healthy Japanese children (152 boys and 146 girls; age range: 5.6–18.4 years). The details related to their initial recruitment have been reported in our previous study (Taki et al., 2010). Using an advertisement issued to local schools, we recruited only right-handed children with no history of malignant tumors or head traumas involving the loss of consciousness. Using the Edinburgh Handedness Inventory, we confirmed that all participants were right handed (Oldfield, 1971). As per the Declaration of Helsinki (1991), after explaining the purpose and procedures of the study, written informed consent was obtained from each participant and his/her parent prior to MR scanning. Approval for this study was obtained from the Institutional Review Board of Tohoku University.

All participants performed the age-appropriate version of Wechsler intelligence scales (Japanese version of WAIS, Third Edition, or WISC, Third Edition). Then, we selected participants whose intelligence was measured using the WISC-III. This yielded 289 children with an age range of 5–16 years. Because of issues with the quality of imaging data or lack of effective data for psychological variables, the analyses that were performed with 277 participants (138 boys and 139 girls) are as follows.

### 2.2 Assessments of psychological variables

On the same day of scanning, intelligence was measured using the WISC-III, administered by trained examiners. Using the raw scores of participants, we calculated three IQ scores (FSIQ, VIQ, and PIQ) and four factor indexes (VC, PO, FD, and PS) for each participant.

Socio-economic status was also measured using the three questions as follows. One was an enquiry relating to family annual income, as reported in our previous study (Taki et al., 2010). Annual income data were collected using discrete variables: 1, annual income <US$ 20,000 (the currency exchange rate was set at US$ 1 = 100 yen); 2, annual income US$ 20,000–40,000; 3, annual income US$ 40,000–60,000; 4, annual income US$ 60,000–80,000; 5, annual income US$ 80,000–100,000; 6, annual income US$ 100,000–120,000; 7, annual income ≥US$ 120,000. The values 1–7 were used in subsequent regression analyses. The other two questions related to the highest educational qualification of both parents. There were eight options: 1, elementary school graduate or below; 2, junior high school graduate; 3, high school graduate; 4, graduate of a short-term course completed after high school (such as junior college); 5, university graduate; 6, Masters degree; and 7, Doctorate, and each choice was converted into the number of years taken to complete the qualification in a normal manner in the Japanese education system (1, 6 years; 2, 9 years; 3, 12 years; 4, 14 years; 5, 16 years; 6, 18 years; 7, 21 years). The average of converted values for each parent was used in the analyses. This protocol followed the standard approach used by the Japanese government for evaluating socio-economic status.

### 2.3 Behavioral data analysis

Behavioral data was analyzed using SPSS version 20 (IBM, Japan). In the unsupervised classification approach, we applied K-means cluster analysis to classify the participants along with the pattern of their factor index scores. Following the procedure applied by Thaler et al. (2013), we increased the number of cluster solutions from three until reaching the point where a minimum of one cluster that contained less than 10% of the entire sample appeared. Thereafter, a supervised classification approach, discriminant function analysis, was performed with factor index scores as predictors and cluster membership as criterion variables to examine the classification accuracy of the cluster solution.

Once the optimal solution was identified, we performed a two-way ANOVA to examine the effects of the factors such as clusters and the scores of WISC-III (VIP, PIQ, VC, PO, FD, and PS). The test of simple main effect was performed when an interaction between the factors was observed. We then performed a one-way ANOVA to examine differences with respect to FSIQ and age and years of education of father and mother. Results with a threshold of *P* < 0.05 were considered statistically significant.

### 2.4 Image acquisition

All images were collected using a 3-T Philips Intera Achieva scanner. Three-dimensional (3D) high-resolution T1-weighted images (T1WI) were collected using a magnetization-prepared rapid gradient-echo sequence. The parameters are as follows: 240 × 240 matrix, TR = 6.5 ms, TE = 3 ms, TI = 711 ms, FOV = 24 cm, 162 slices, 1.0 mm slice thickness, and scan duration of 8 min and 3 s.

### 2.5 Pre-processing and analysis of structural data

The 3D T1-weithted MRI datasets were analyzed using voxel-based morphometry. The pre-processing of structural data was performed using the statistical parametric mapping software (SPM8; Wellcome Department of Cognitive Neurology, London, UK) implemented in Matlab (Mathworks Inc., Natick, MA, USA). Using the new segmentation algorithm implemented in SPM8, T1-weighted structural images of each individual were segmented into six tissues. The gray matter tissue probability map (TPM) used in this procedure was manipulated from maps implemented in the software for the signal intensities of the voxels that were (gray matter tissue probability of the default tissue gray matter TPM + white matter tissue probability of the default TPM) <0.25 to become 0. When compared with the default gray matter TPM, when this manipulated gray matter TPM is used, the dura matter is less likely to be classified as gray matter, without other substantial segmentation problems. In this new segmentation process, default parameters were used, except for affine regularization that was performed using the International Consortium for Brain Mapping template for East Asian brains. We then proceeded into diffeomorphic anatomical registration through the exponentiated lie algebra (DARTEL) registration process implemented in SPM8. In this process, we used DARTEL-imported images of the 5 TPM of gray matter created using the aforementioned new segmentation process. First, the template for the DARTEL procedures was created using the T1WI data from all participants. The resulting images were then spatially normalized to the Montreal Neurological Institute (MNI) space to obtain images with 1.5 mm × 1.5 mm × 1.5 mm voxels. Subsequently, all images were smoothed by convolving them with an isotropic Gaussian kernel of 10 mm full-width at half-maximum.

### 2.6 Statistical analysis

Statistical analyses of imaging data were performed with SPM8. In the analyses of rGMV, we included only voxels that showed rGMV values >0.10 in all participants. The primary purpose for using this type of threshold was to cut the periphery of the gray matter area and to effectively limit the area for analysis to areas that are likely to be gray matter.

To examine brain regions that were larger or smaller in certain subtypes than others, we conducted ANOVA using FWE *P* < 0.05 with uncorrected *P* < 0.001 at voxel level, and there was no significant results. However, we could find significant voxels with a more liberal threshold (uncorrected *P* < 0.001 without FWE correction). Therefore we set contrasts as follows. We set 5 for the target subtype and −1 for other subtypes to examine brain regions that were larger than other subtypes. We then set −5 for the target subtype and 1 for other subtypes to examine brain regions that were smaller than other subtypes. Age, gender, FSIQ, and total intracranial volume were treated as covariates, and rGMV at each voxel was treated as a dependent variable. The statistical threshold was set at *P* < 0.05, corrected at the non-isotropic adjusted cluster level, with an underlying voxel level of *P* < 0.001. Because there were 12 multiple comparisons, we corrected *P* values using Bonferroni correction. The corrected *P* value was set at *P* < 0.0042 (derived from *P* = 0.05/12 contrasts).

## 3 Results

### 3.1 Cluster analysis

The six-cluster solution was the largest solution, including samples that were more than 10% of the entire sample in each cluster. Then, four factor index scores of WISC-III were used as the input for discriminant function analysis to establish their classification rate for predicting clusters. Factor index scores accurately predicted 96.8% of the six-cluster cases. The number of subtypes was similar with previous findings on typically developing children (Donders, 1996, Glutting et al., 1994). Thus, the six-cluster solution was used in the analyses as follows.

### 3.2 Group analysis

**Fig. 1:**
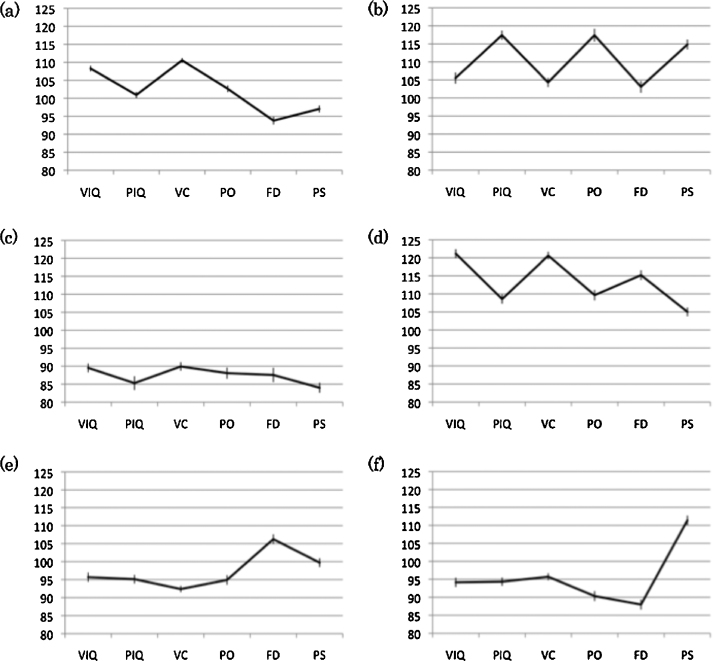
Within-group difference for each subtype. Cognitive patterns of (a) C1, (b) C2, (c) C3, (d) C4, (e) C5, and (f) C6 are indicated. Error bars represent standard errors. VIQ: verbal IQ, PIQ: performance IQ, VC: verbal comprehension, PO: perceptual organization, FD: freedom from distractibility, and PS: processing speed.

The two-way ANOVA demonstrated significant effects of subtype (*F*(5, 1897) = 384, *P* < .001) and WISC-III scores (*F*(6, 1897) = 5.7, *p* < 0.001). The interaction between clusters and scores was also significant (*F*(30, 1897) = 23.9, *P* < 0.001). From the results of the simple main effect analysis of cluster discrepancies, cluster 1 (C1) featured a higher VIQ score than PIQ (*P* < 0.001), the highest VC score among the scores of other factor index scores (*P* < 0.001), and a higher PO score than FD (*P* < 0.001) and PS (*P* < 0.01). The discrepancy between the scores of FD and PS was not significant. In cluster 2 (C2), the PIQ score was superior to VIQ (*P* < 0.001), and the scores of PO and PS were higher than VC (*P* < 0.001) and FD (*P* < 0.001). In cluster 3 (C3), the discrepancy of all six scores were not significant with respect to each other. In cluster 4 (C4), VIQ score was higher than PIQ (*P* < 0.001), the VC score was the highest among the scores of other factor indexes (PO and PS: *P* < 0.001, FD: *P* < 0.05), and the FD score was higher than PO scores (*P* < 0.05) and PS scores (*P* < 0.001). In cluster 5 (C5), although the discrepancy between VIQ and PIQ was not significant, the FD score was the highest among the scores of other 3 factor indexes (VC, PO: *P* < 0.001, PS: *P* < 0.01), and the PS score was higher than VC (*P* < 0.001). In cluster 6 (C6), the discrepancy between VIQ and PIQ was also not significant, the PS score was the highest among the scores of other 3 factor indexes (VC, PO, FD: *P* < 0.001), and the VC score was higher than the score of FD (*P* < 0.01). These results are indicated in Fig. 1.

The one-way ANOVA demonstrated significant subtype differences in age (*F*(5, 271) = 4.25, *P* < 0.05). Post-hoc multiple comparison with Bonferroni correction revealed that children in C1 and C6 were older than those in cluster 5 (both pairs; *P* < 0.05). The differences of years of education of parents were not significant (father; *F*(5, 270) = 0.26, n.s., mother; *F*(5, 271) = 1.19, n.s.) (Table 1).

**Table 1:** Age, years of education of parents, and scores of WISC of six cluster subtypes. Average values are given with standard deviations in parentheses for age, years of education, and four WISC scores. Statistical threshold was set at *P* < 0.05 with Bonferroni correction.

Cluster	1		2		3		4		5		6		Statistical significance
*n*	64		39		31		52		50		41		
Age	11.9	(2.7)	10.2	(2.8)	11.2	(2.9)	10.7	(2.8)	10.0	(3.1)	12.0	(3)	1 > 5, 6 > 5
Years of education (father)	14.7	(2.5)	14.9	(2)	14.7	(1.7)	14.7	(2.2)	14.5	(2.1)	14.6	(2.3)	n.s.
Years of education (mother)	13.8	(1.8)	13.9	(1.4)	14.1	(1.6)	14.1	(2)	13.7	(1.7)	13.4	(1.4)	n.s.
Scores of WISC													
FSIQ	105.3	(5.3)	112.4	(7.9)	86.3	(6.6)	116.8	(7.8)	95.0	(6.3)	93.9	(6.3)	
VIQ	108.2	(6.4)	105.5	(9.8)	89.5	(6.9)	121.2	(8.9)	95.7	(9.2)	94.2	(8.3)	4 > 1 = 2 > 5 = 6 = 3
PIQ	100.9	(6.8)	117.4	(8)	85.3	(10.7)	108.6	(9.2)	95.1	(8.5)	94.3	(7.3)	2 > 4 > 1 > 5 = 6 > 3
VC	110.6	(7.5)	104.3	(11.1)	89.9	(8.7)	120.6	(10.5)	92.4	(9.5)	95.7	(8.8)	4 > 1 > 2 > 3 = 6 = 5
PO	102.6	(8.6)	117.4	(10.3)	88.1	(11.2)	109.7	(9.8)	94.9	(9.5)	90.3	(8.8)	2 > 4 > 1 > 5 > 3, 5 = 6, 3 = 6
FD	93.8	(7.7)	103.1	(8.7)	87.6	(7.8)	115.2	(8.8)	106.2	(8.7)	88.0	(8.4)	4 > 2 > 5 > 1 > 6 = 3
PS	97	(8)	114.8	(9.6)	84.0	(7.4)	105.0	(11)	99.7	(9)	111.4	(8.2)	2 = 6 > 4 > 1 = 5 > 3

FIQ, full scale IQ; VIQ, verbal IQ; PIQ, performance IQ; VC, verbal comprehension; PO, perceptual organization; FD: freedom from distractibility; PS, processing speed. “ = ” means no significant among subtypes.

### 3.3 Imaging results

**Fig. 2:**
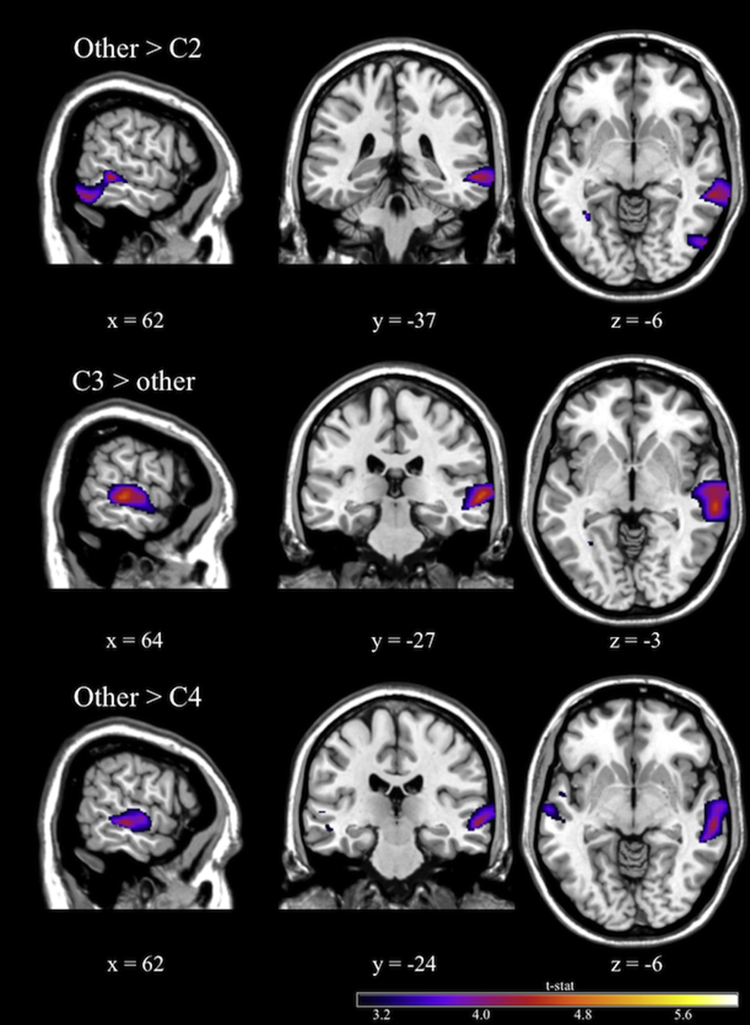
Difference in rGMV among clusters. Middle and superior temporal gyri were significantly larger in C3 than in other clusters (middle). These regions were significantly smaller in C2 (upper) and marginally smaller in C4 (lower) than in other clusters. For multiple comparisons, *P* value was set at *P* < 0.0042 with Bonferroni correction.

The right middle temporal and superior temporal gyri were significantly larger in C3 than in the other five clusters (see Fig. 2, middle panel). The inferior and middle temporal gyri were significantly smaller in C2 (Fig. 2, upper panel) than in other clusters. Moreover, middle and superior temporal gyri were marginally smaller in C4 than in the other five clusters (Fig. 2, lower panel). MNI coordinates, cluster size, and other statistical information are shown in Table 2.

**Table 2:** Brain regions and MNI coordinates showing significant differences among subtypes. Statistical threshold was set at *P* < 0.05, with FWE corrected in non-isotropic adjusted cluster level. For multiple comparisons, *P* value was set at *P* < 0.0042 with Bonferroni correction.

Contrasts	Cluster Size	Peak voxel				*P* value	Anatomical region
	(voxels)	MNI Coordinates			*T* score		
		*X*	*Y*	*Z*			
C3 – other clusters	2135	64	−27	−3	4.6	0.001	Middle temporal gyrus
		56	−15	0	4.56		Superior temporal gyrus
		56	−31	−8	4.14		Middle temporal gyrus
Other clusters – C2	1527	63	−54	−21	4.24	0.004	Inferior temporal gyrus
		56	−72	−9	4.17		Middle occipital gyrus
		62	−37	−6	4.13		Middle temporal gyrus
Other clusters – C4	1417	62	−24	−6	4.49	0.006^†^	Middle temporal gyrus
		56	−15	0	3.97		Superior temporal gyrus
		51	−33	−9	3.67		Middle temporal gyrus

^†^Marginally significant.

## 4 Discussion

From the results of cluster analysis, we found six subtypes from the cognitive patterns of the scores of factor indexes based on the WISC-III. In these subtypes, C1, C2, and C4 had average performance levels. C1 showed a higher score in VIQ than in PIQ and the highest score in VC. In C2 and C4, the factor index score pattern was reversed; C2 showed higher scores in PO and PS but C4 showed higher scores in VC and FD. C3, C5, and C6 showed relatively lower scores in IQ and factor index scores. However, C5 presented higher scores in FD, and C6 presented higher scores in PS. This is the first study to investigate the relationship between individual cognitive patterns and brain structure. The hypotheses that prefrontal, temporal, and parietal cortices might be related to certain cognitive profile subtypes were partially proven. When compared with other subtypes, there was larger gray matter volume in C3 and smaller volume in C2 and C4 in the right middle temporal gyrus. In addition, the cluster of the right middle temporal gyrus covered posterior regions, including the superior temporal gyrus. However, we did not find any significant relationships between cognitive patterns and prefrontal and parietal regions.

When considering the brain imaging results, the larger volume of the right middle temporal gyrus in C3 indicated generally lower performance in VIQ, PIQ, and factor index scores in WISC than other subtypes. This result is consistent with previous neuroimaging studies of brain maturation in typically developing children. Previous studies on the maturation of brain structure consistently showed a reduction in gray matter volume in the frontal, temporal, and parietal cortices along with the age (Chugani, 1998, Gogtay et al., 2004, Taki et al., 2011). In particular, the peak volume of the temporal cortex occurred between 10 and 12 years (Taki et al., 2011). Consistent with these results, C3 children appeared under-developed when compared with children in other subtypes. Conversely, C2 and C4 children had smaller volume in the right middle temporal gyrus and higher performance levels in VIQ and PIQ than other subtypes and were interpreted to suggest that brain structure was more mature in these children than the other subtypes.

However, previous studies that focused on the relationship between cognitive performance and rGMV showed a positive correlation between FSIQ and certain brain regions, including orbitofrontal and medial frontal regions and the cingulate cortex (Frangou et al., 2004, Gong et al., 2005, Haier et al., 2004, Lee et al., 2005). These results are inconsistent with the results of this study. This might be because of the difference of the sample population. The abovementioned previous studies mainly focused on participants who were older than those in this study. Other brain regions, especially frontal regions, matured later than posterior regions; thus, a positive correlation between FSIQ and rGMV appeared to be revealed in these studies. Moreover, the performance level of the participants in the previous studies was relatively higher than that in this study. Shaw et al. (2006) revealed that the enlargement of brain cortical thickness in the superior cognitive performance group lasted longer than that in higher and average cognitive performance groups in the right superior frontal, medial prefrontal, and middle temporal gyri. Therefore, higher performance populations were considered to tend to show a positive correlation between IQ and brain structure.

Some previous imaging studies revealed that the right middle temporal gyrus and adjacent regions (including the superior temporal gyrus) were responsible for language ability, including higher-order auditory and semantic processing (Chou et al., 2006, Friederici et al., 2009, Makris et al., 2013, Willems et al., 2009). Other studies clarified the relationship between arithmetic ability and working memory maintenance (Park et al., 2011, Starke et al., 2013). Moreover, Margolis et al. (2013), who investigated the neural underpinnings of discrepancy in VIQ and PIQ, revealed that the degree of discrepancy was correlated with the cortical thickness of anterior and posterior cortices, including medial prefrontal, superior temporal, and occipital cortices. These results suggest that these regions account for a large portion of the overall variance in the magnitude of the discrepancy between VIQ and PIQ. Thus, the right middle temporal gyrus plays an important role in cognitive development.

Cluster analysis of the patterns of factor index scores indicated subtypes with distinctive patterns. Previous studies did not examine intragroup discrepancies using statistical techniques. This study presented detailed findings regarding individual cognitive discrepancies in typically developing children. From the viewpoint of performance, lower- (C3, 5, and 6) and average-level subtypes were found (C1, 2, and 4), but higher-level subtypes (above 1SD in all four factor index scores) were not. However, the latter result was revealed by Donders (1996). The result of significant age differences among subtypes in this study also differed from that of Donders (1996). Children were significantly younger in C5 than in C1 and C6; this represents that individual cognitive pattern might change along with the age. Moreover, we did not find any significant difference in the years of education of parents, whereas Donders (1996) found that one-third of the children in the subtype with above-average performance came from families where parents had sixteen or more years of education and 40% of children in the below-average performance subtype came from families where parents had less than twelve years of education. The level of cognitive performance was considered to differ regardless of parents’ years of education. Alternatively, it might be because of differences in sampling. Donders (1996) used a standardization sample of WISC-III and examined a large sample (*n* = 2200). In our study, samples had some bias, and the years of education were relatively high.

There are some limitations in our study. First, brain regions that previously reported to show a significant relationship with cognitive performance, such as the frontal and parietal gyri, were not significantly related to cognitive patterns in this study. This inconsistency is because of differences in sample age, level of cognitive performance, or scores used in imaging analysis. Almost all previous imaging studies used FSIQ as an independent variable in multiple regression. Therefore, the difference regarding general cognitive performance (e.g., FSIQ) appeared to be represented by the frontal and parietal regions, with more detailed differences in cognitive patterns modulated by more focal regions. Second, we did not find a subtype with above-average performance. This might be because of the smaller sample size of this study. Previous studies revealing cognitive patterns in typically developing children examined a larger sample size, such as a standardization sample.

In conclusion, this study revealed individual differences in cognitive patterns and brain structure in typically developing children. We found six subtypes dependent on patterns of four factor index scores. These subtypes were differentiated by the level of performance (below average or average) and performance patterns. A subtype with below-average general cognitive performance levels showed larger rGMV in the right middle temporal gyrus than in other subtypes. Moreover, two subtypes with relatively high cognitive performance showed smaller rGMV in the right middle temporal gyrus than in other subtypes. This suggests that individual differences in cognitive patterns appears to be modulated by this region. This is the first study to investigate the relationship between individual differences in cognitive patterning and brain structure. The profile analysis of cognitive testing is useful for a better understanding of cognitive strengths and weaknesses of not only typically developing children but also children with neurodevelopmental disorders, such as autism spectrum disorder, ADHD, or learning disorders. The results of this study will be helpful in investigating individual differences and brain structure in children with these disorders.

## Conflict of interest

We wish to confirm that there are no known conflicts of interest associated with this publication and there has been no significant financial support for this work that could have influenced its outcome.
